# Supplementary material for: Genome-wide microhomologies enable precise template-free editing of biologically relevant deletion mutations
Source: Nat Commun. 2019 Oct 24;10:4856. doi: 10.1038/s41467-019-12829-8 (PMC6813315; doi:10.1038/s41467-019-12829-8)
Supplement: Supplementary file 1 — Supplementary Information [file 41467_2019_12829_MOESM1_ESM.pdf]

SUPPLEMENTARY INFORMATION

Genome-wide microhomologies enable precise template-free editing of biologically relevant deletion mutations

Grajcarek et al.

This file includes:

Supplementary Figures 1-7

Supplementary Tables 1-7

SUPPLEMENTARY FIGURES

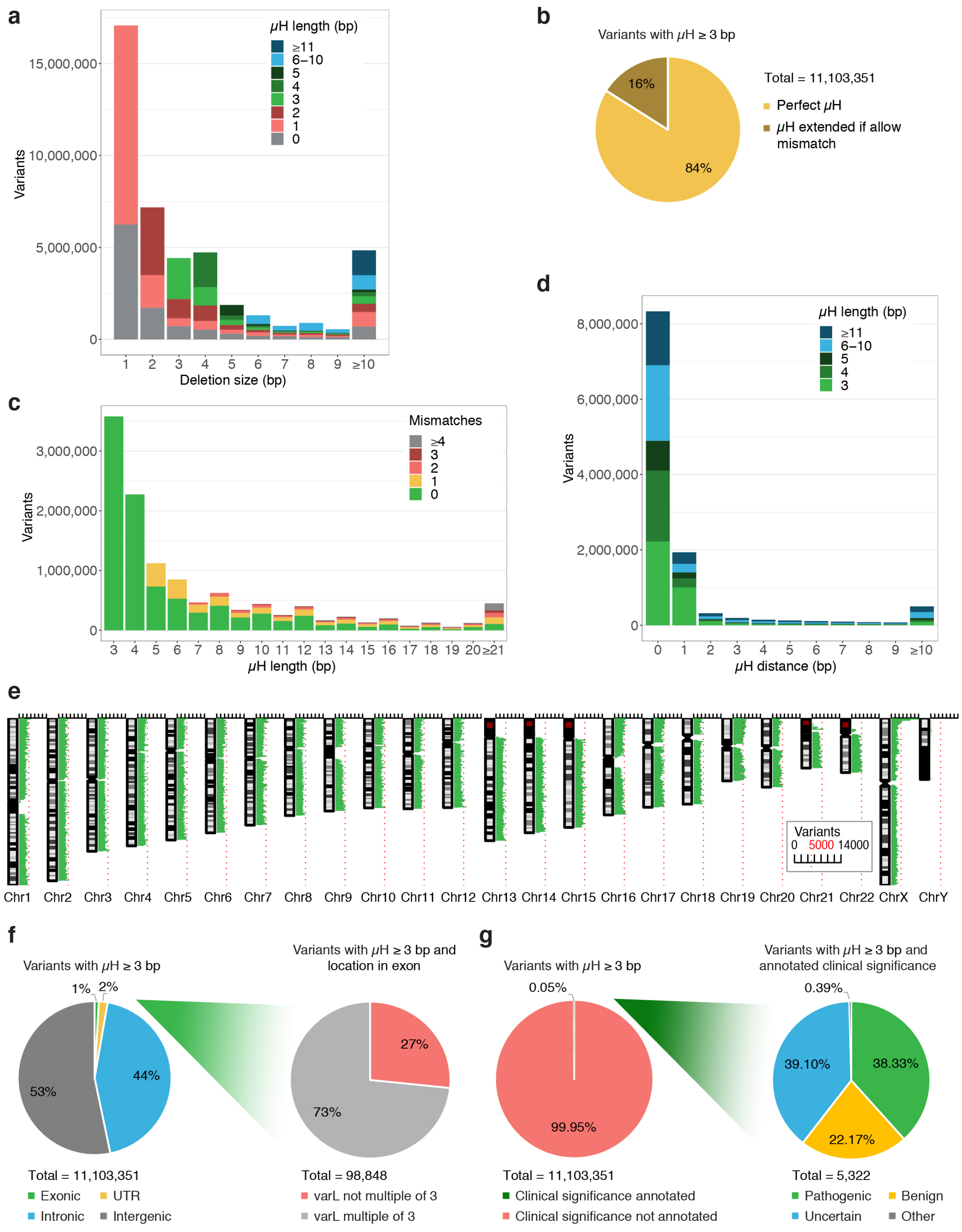

Supplementary Figure 1. Analysis of human deletion alleles flanked by microhomologies (μHs).

(a) Deletion variants plotted by deletion size with μH length indicated by fill color.

(b) Percentage of μH-flanked deletion variants for which the μH length would be extended, if one consecutive mismatch is allowed.

- **(c)** Number of deletion variants with one continuous stretch of ≥ 3 bp μH by μH length with number of mismatches indicated by fill color.
- **(d)** Distribution of deletion variants with one continuous stretch of ≥ 3 bp μH by μH distance with μH length indicated by fill color.
- **(e)** Number of μH-flanked deletion variants per Mbp across all chromosomes.
- **(f)** Genomic location of μH-flanked deletion variants and proportion of exonic variants resulting in a frameshift on the right; un-translated region (UTR); variant length (varL).
- **(g)** Percentage of μH-flanked deletion variants with annotated clinical significance and number of variants per category of clinical significance on the right.

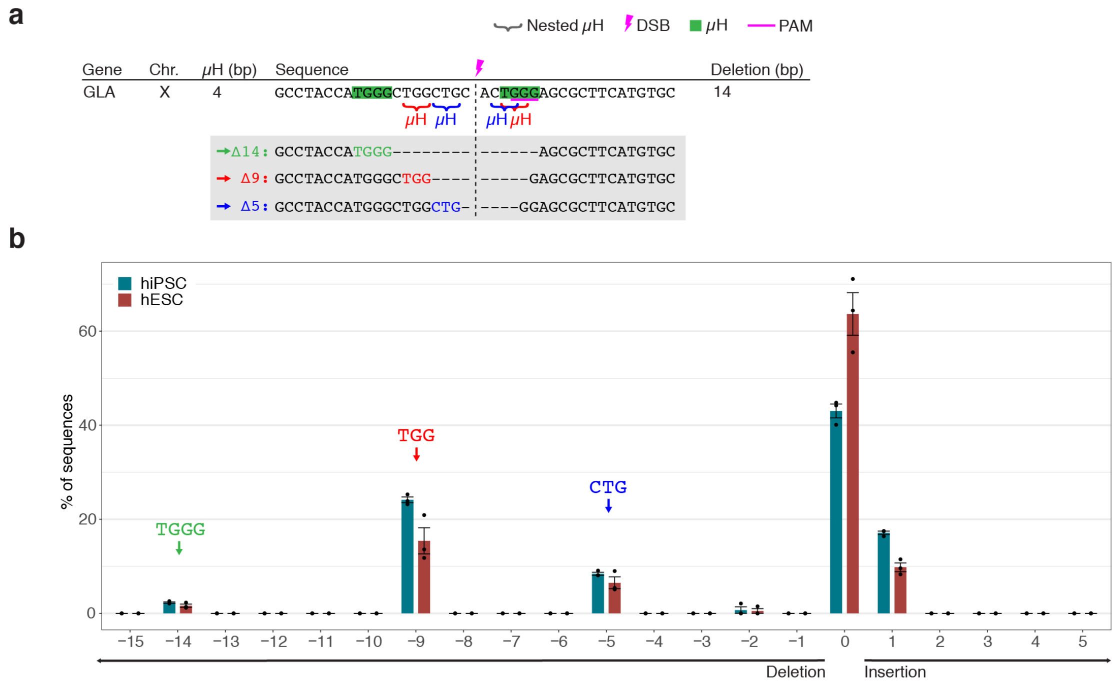

Supplementary Figure 2. Nested µHs decrease efficiency of targeted mutation outcome.

(a) Target variant containing nested µH in GLA gene. Possible resulting deletion sizes are shown in grey box. µH (green), nested µHs (red, blue), DSB location (pink bolt), SpCas9 PAM (underline).

(b) Percentage of RNP transfected 1383D6 hiPSCs or H1 hESCs cell populations carrying insertions or deletions in target GLA-14bpDel locus. In hiPSCs, the target 14 bp deletion ("TGGG" µH) accounts for 2% of the alleles, while the nested 9 bp deletion ("TGG" µH) and the 5 bp deletion ("CTG" µH) comprise 24% and 8% of the alleles respectively. Means ± s.e.m. for n = 3 biological replicates.

a

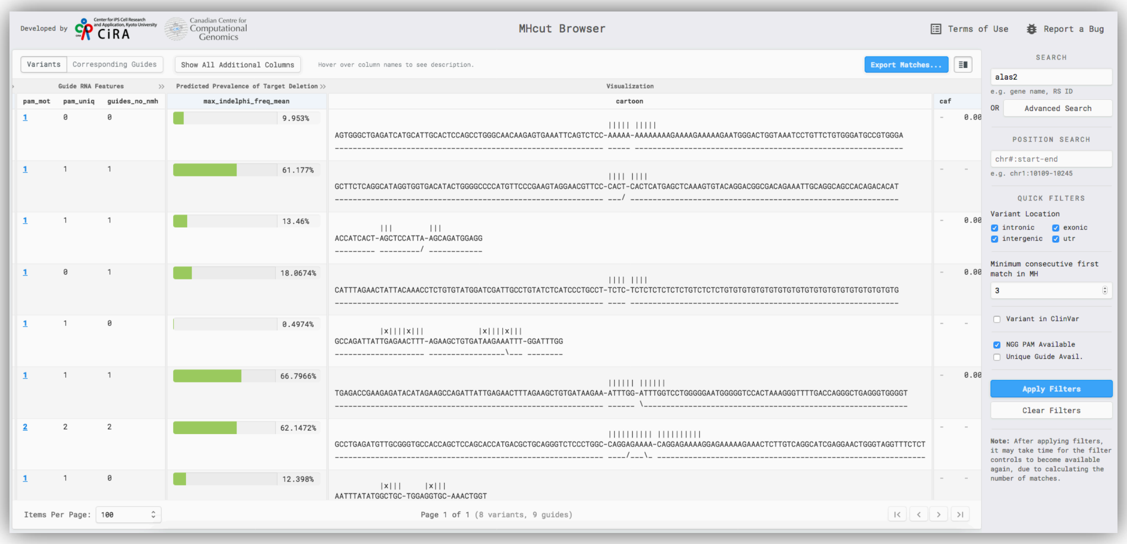

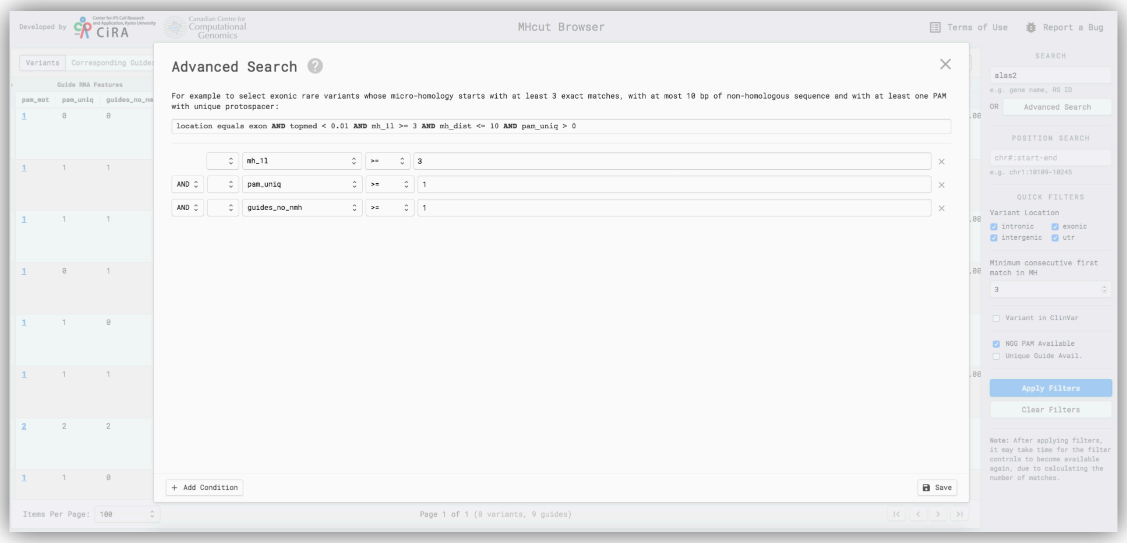

Supplementary Figure 3. The output of the MHcut tool with additional filter options can be accessed online in the MHcut browser data portal.

(a) MHcut browser quick search features enable for example direct selection of gene of interest and microhomology length.

(b) The advanced search allows to filter all available data columns for freely selectable criteria with different logic statements e.g. “contains”, “=”, and “≥”.

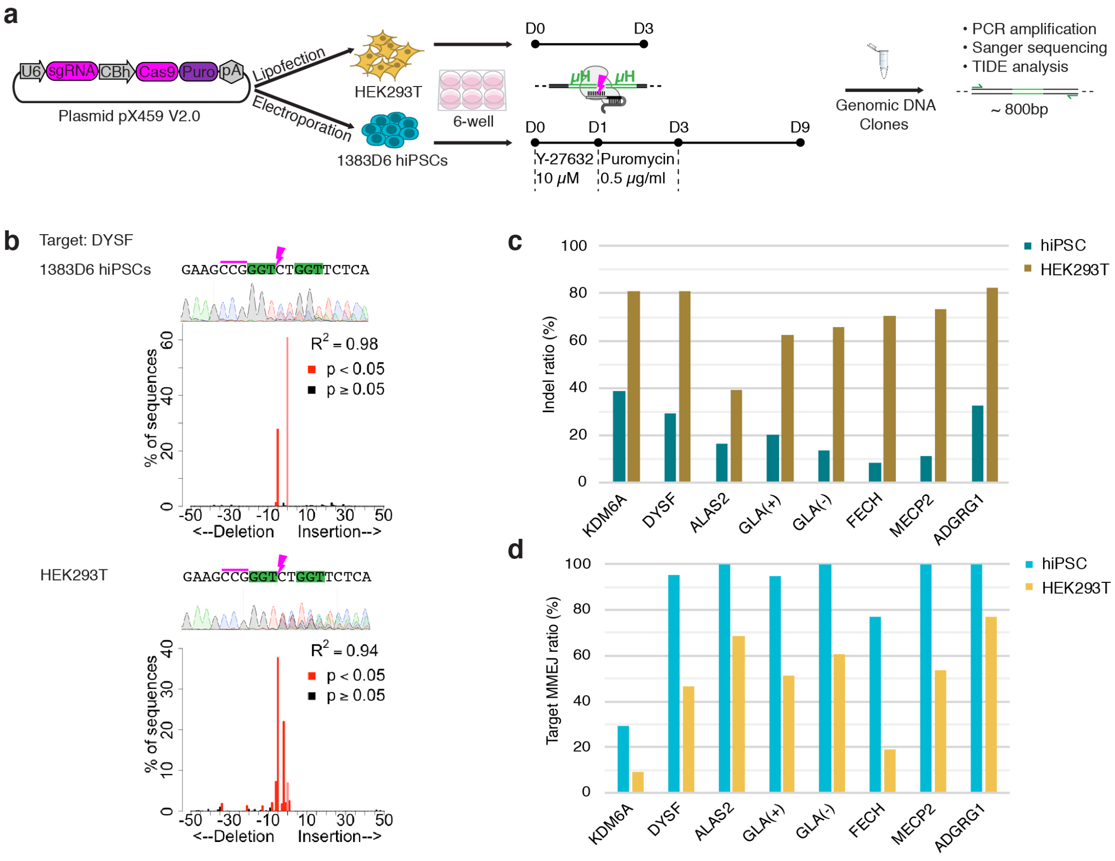

Supplementary Figure 4. Target μH-flanked deletion mutations can be recreated with high precision in hiPSCs and HEK293T cells.

- **(a)** Schematic of the experimental method used to create MMEJ deletion alleles in 1383D6 hiPSCs and HEK293T cells.
- **(b)** Percentage of transfected hiPSC or HEK293T cell populations carrying insertions or deletions in target DYSF locus. $p<0.05$, two-tailed *t*-test.
- **(c)** Overall ratio of indel mutations found in the transfected hiPSC or HEK293T cell populations.
- **(d)** Ratio of the target MMEJ outcome among total indels.

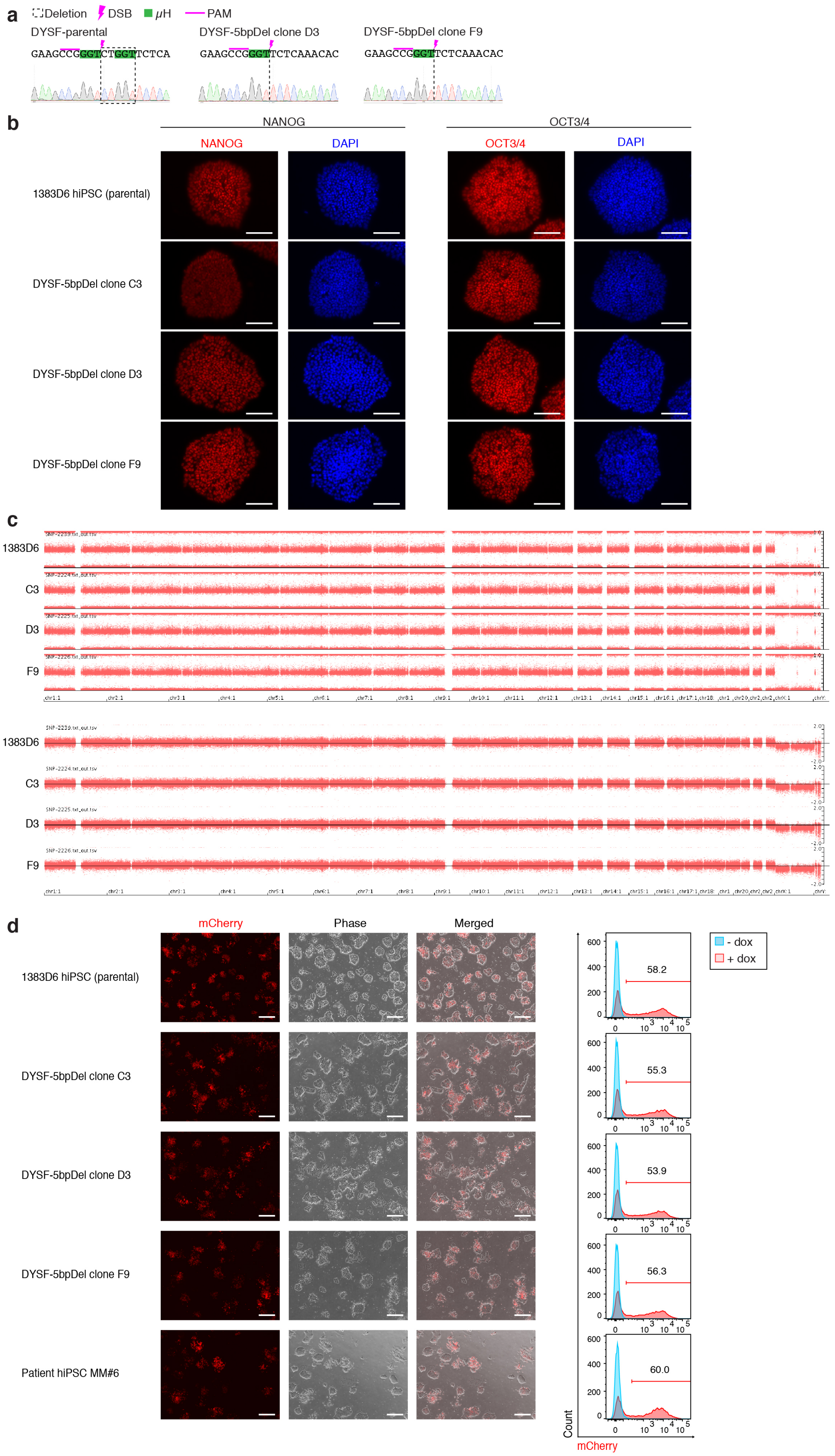
a
Deletion
DSB
μH
PAM
DYSF-parental
GAAGCCGGGTCTGGTTCTCA
DYSF-5bpDel clone D3
GAAGCCGGGTTCTCAAACAC
DYSF-5bpDel clone F9
GAAGCCGGGTTCTCAAACAC
b
NANOG
OCT3/4
NANOG
DAPI
OCT3/4
DAPI
1383D6 hiPSC (parental)
DYSF-5bpDel clone C3
DYSF-5bpDel clone D3
DYSF-5bpDel clone F9
c
1383D6
C3
D3
F9
1383D6
C3
D3
F9
d
mCherry
Phase
Merged
1383D6 hiPSC (parental)
DYSF-5bpDel clone C3
DYSF-5bpDel clone D3
DYSF-5bpDel clone F9
Patient hiPSC MM#6
- dox
+ dox
58.2
55.3
53.9
56.3
60.0
Count
mCherry

Supplementary Figure 5. Characterization of hiPSC DYSF-5bpDel clones.

(a) Sequence verification of a precise 5 bp deletion mutation in DYSF. Deletion (dotted line), DSB location (pink bolt), μH (green), SpCas9 PAM (underline).

(b) Immunostaining for the pluripotency markers NANOG and OCT3/4 in undifferentiated DYSF-5bpDel clones and parental hiPSCs. Right panels show DAPI staining. Scale bar indicates 100 μm.

(c) Karyogram showing all chromosomes of undifferentiated DYSF-5bpDel clones and parental 1383D6 hiPSCs. B Allele Frequency (top) and Log R Ratio (bottom) of SNP array (v1.2) analysis detect no large CNVs.

(d) Analysis of MYOD induction as indicated by mCherry expression in transfected parental hiPSC, three derived DYSF-5bpDel clone and patient hiPSC populations after 48h dox treatment. Fluorescent and phase-contrast images on the left; FACS quantification of mCherry positive population on the right; scale bar indicates 500 μm; dox treated population (red), dox negative population (blue).

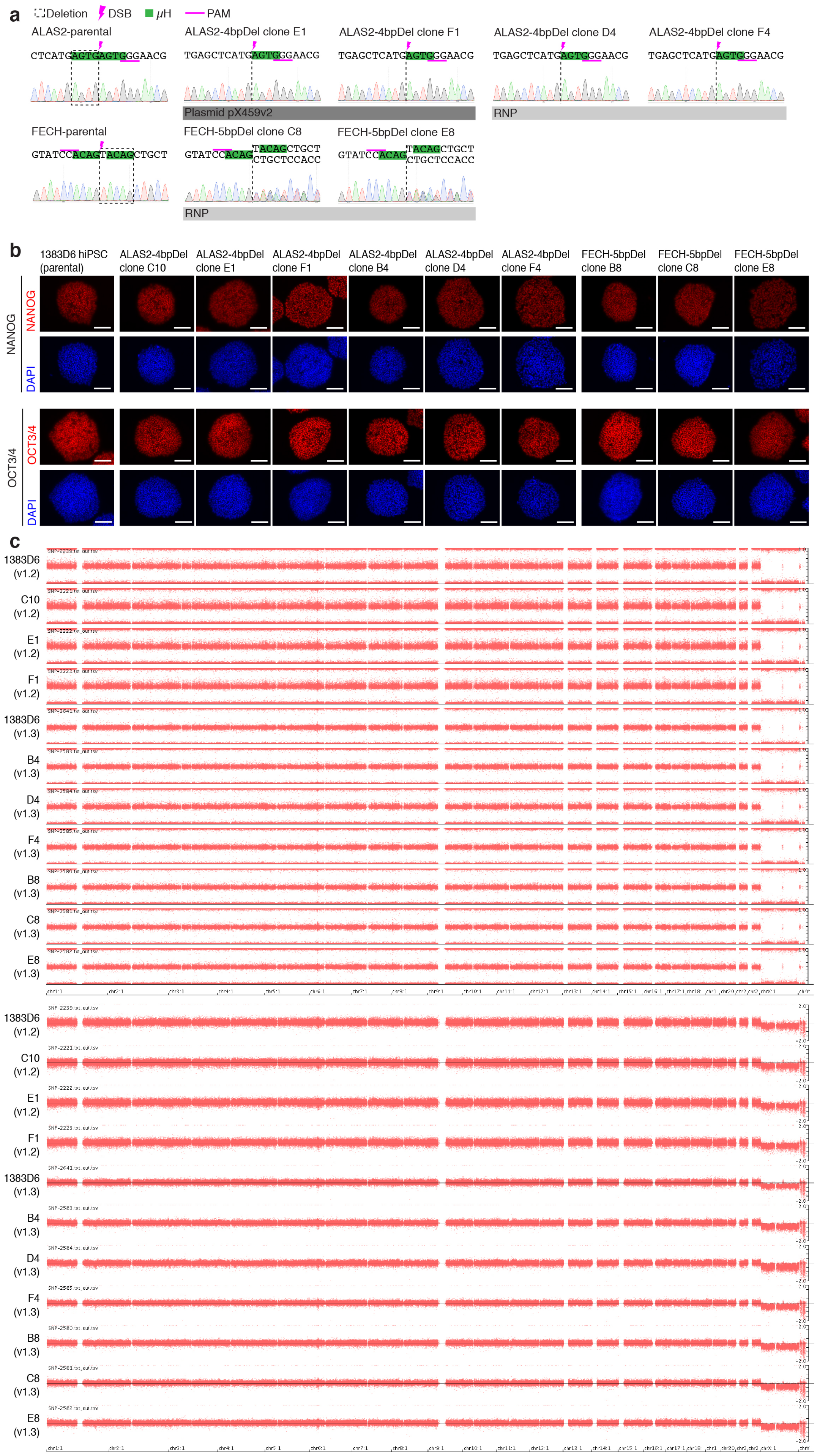

a
Deletion
DSB
μH
PAM
ALAS2-parental
CTCATGAGTGAGTGGGAACG
ALAS2-4bpDel clone E1
TGAGCTCATGAGTGGGAACG
ALAS2-4bpDel clone F1
TGAGCTCATGAGTGGGAACG
ALAS2-4bpDel clone D4
TGAGCTCATGAGTGGGAACG
ALAS2-4bpDel clone F4
TGAGCTCATGAGTGGGAACG
Plasmid pX459v2
RNP
FECH-parental
GTATCCACAGTACAGCTGCT
FECH-5bpDel clone C8
GTATCCACAG
TACAGCTGCT
CTGCTCCACC
FECH-5bpDel clone E8
GTATCCACAG
TACAGCTGCT
CTGCTCCACC
RNP
b
1383D6 hiPSC (parental)
ALAS2-4bpDel clone C10
ALAS2-4bpDel clone E1
ALAS2-4bpDel clone F1
ALAS2-4bpDel clone B4
ALAS2-4bpDel clone D4
ALAS2-4bpDel clone F4
FECH-5bpDel clone B8
FECH-5bpDel clone C8
FECH-5bpDel clone E8
NANOG
NANOG
DAPI
OCT3/4
OCT3/4
DAPI
c
1383D6 (v1.2)
C10 (v1.2)
E1 (v1.2)
F1 (v1.2)
1383D6 (v1.3)
B4 (v1.3)
D4 (v1.3)
F4 (v1.3)
B8 (v1.3)
C8 (v1.3)
E8 (v1.3)

d

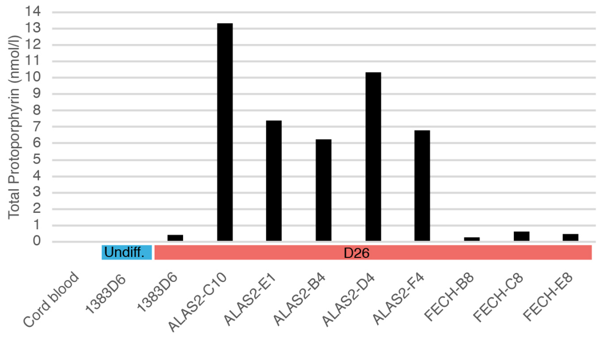

Supplementary Figure 6. Characterization of hiPSC ALAS2-4bpDel and FECH-5bpDel disease model clones.

(a) Sequence verification of a precise 4 bp deletion mutation in ALAS2 and a 5 bp deletion in FECH generated by either plasmid or RNP transfection. Deletion (dotted line), DSB location (pink bolt), µH (green), SpCas9 PAM (underline).

(b) Immunostaining for the pluripotency markers NANOG and OCT3/4 in ALAS2-4bpDel and FECH-5bpDel clones and in parental hiPSCs. Right panels show DAPI staining. Scale bar indicates 100 µm.

(c) Karyogram showing all chromosomes of undifferentiated ALAS2-4bpDel and FECH-5bpDel clones and parental 1383D6 hiPSCs. B Allele Frequency (top) and Log R Ratio (bottom) of SNP array (v1.2 and v1.3) analysis detect no large CNVs.

(d) Total protoporphyrin concentration on day 26 of erythroid differentiation culture in ALAS2-4bpDel clones, in FECH-5bpDel clones and in parental hiPSCs; cord blood cells and undifferentiated parental hiPSCs act as controls.

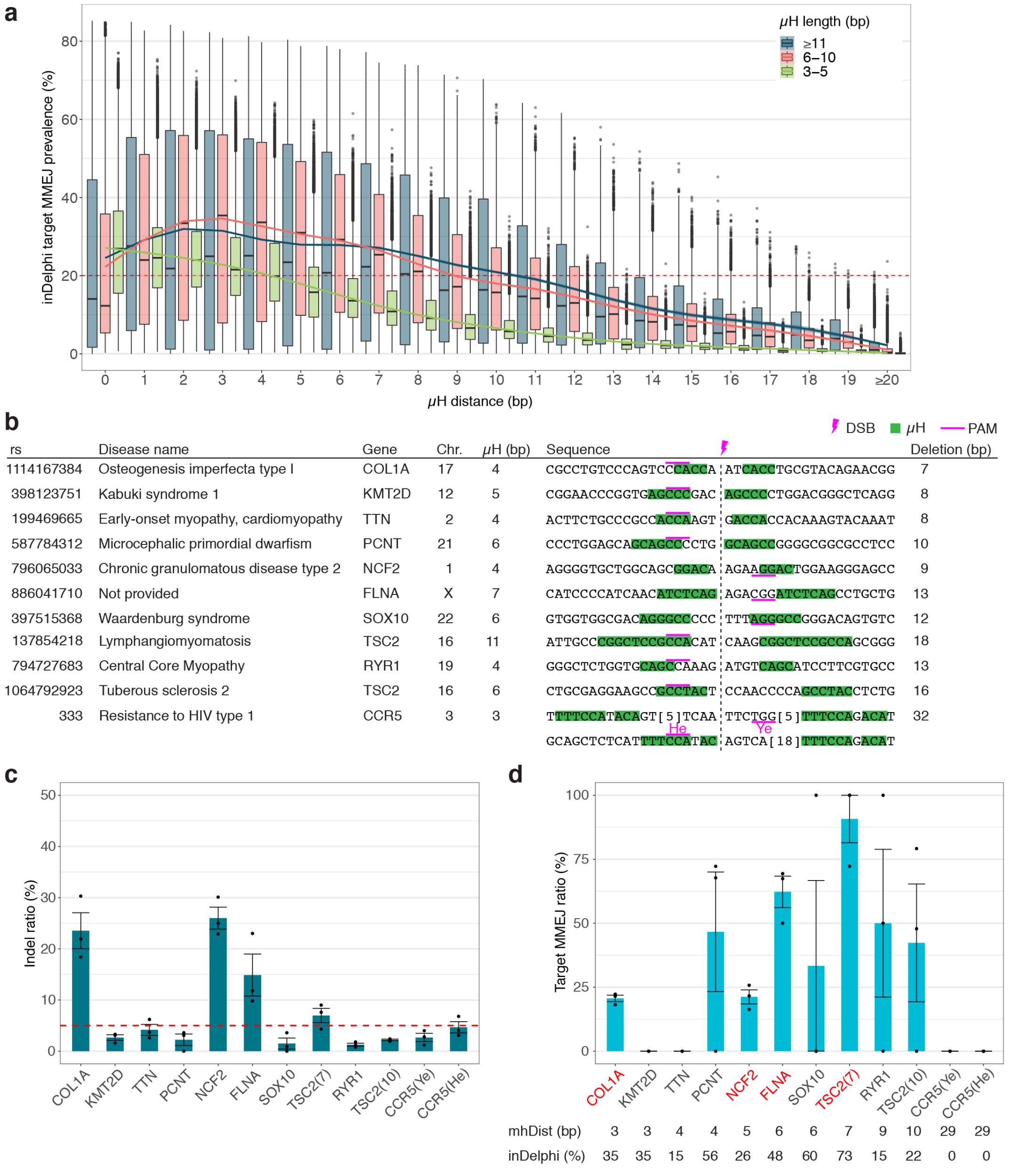

Supplementary Figure 7. Assessing the influence of increasing μH distance on target MMEJ repair outcome efficiency.

(a) Prevalence predicted by inDelphi algorithm (average score from the 5 cell types tested) for all MHcut variants with available Cas9 guide RNA by μH-distance with μH length indicated by fill color. Dotted line represents 20% predicted prevalence.

(b) Selected target variant list. μH (green), DSB location (pink bolt), SpCas9 PAM (underline), Ye (guide RNA targeting CCR5 published by Ye et al., 2014), He

(guide RNA targeting CCR5 presented by Jiankui He at the Second International Summit on Human Genome Editing in 2018).

(c) Overall ratio of indel mutations found in the transfected hiPSC cell populations. For COL1A, FLNA and TSC2 (10) nested μHs of 2 bp size reduce the target MMEJ ratios. TTN has a 4 bp μH outside of the target μHs with a higher GC ratio that reduces the target MMEJ ratio. Dotted line represents 5% overall ratio of indel mutations.

(d) Ratio of the target MMEJ outcome among total indels. Targets in red font have an overall indel ratio above 5%. mhDist (μH distance); inDelphi (predicted prevalence of target MMEJ outcome from inDelphi algorithm); means ± s.e.m. for n = 3 biological replicates.

SUPPLEMENTARY TABLES

Supplementary Table 1. Genotypes of hiPSC FECH clones

Normal	Homozygous mutant	Heterozygous mutant with the other allele normal			Mixed sequence
60	0	26			8
		5 bp Deletion	1 bp Insertion	Other Indels	
		11	12	3	

Supplementary Table 2. MHcut outputs provided in addition to data from ClinVar and dbSNP databases

Category	Name	Explanation
**Variant file**		
IDs	id	Variant ID number assigned by MHcut
Microhomology (µH) features	mh_l	Length of full µH including mismatches (bp)
	mh_1l	Length of first stretch of perfect µH (bp)
	hom	Homology ratio of full µH
	mh_dist	Distance between the µHs based on mh_l (bp)
	mh_1dist	Distance between the µHs based on mh_1l (bp)
	mh_seq_1	Sequence of µH (left side)
	mh_seq_2	Sequence of µH (right side)
	nbmm	Number of mismatches in the µH
	mh_max_cons	Length of longest consecutive stretch of µH within full µH (bp)
	gc	GC content of µH (high GC content is associated with strong µHs[11,14])
	mh_score	µH score calculated by MHcut to decide which flank of the deletion is the stronger µH (number of mh_l + mh_1l)
	flank	Flank configuration chosen by MHcut (1: outer-inner, 2: inner-outer)
Guide RNA features	pam_mot	Number of NGG PAMs in a valid location between the µHs
	pam_uniq	Number of PAMs with a unique protospacer sequence in the genome
	guides_no_nmh	Number of guide RNAs with no nested µH
	guides_min_nmh	Number of nested µHs for guide RNA with the least nested µHs
	max_2cut_dist	Maximum distance between Cas9 cut sites available (bp) (it is possible to use two guide RNAs close to each µH to create large deletions[13])
Predicted Prevalence of Target Deletion	max_indelphi_freq_mean	Maximum prevalence predicted by inDelphi[14] for target deletion (mean of 5 cell types for best guide RNA)
	max_indelphi_freq_mesc	Max. prevalence predicted by inDelphi[14] for target deletion in mESCs
	max_indelphi_freq_u2os	Max. prevalence predicted by inDelphi[14] for target deletion in U2OS
	max_indelphi_freq_hek293	Max. prevalence predicted by inDelphi[14] for target deletion in HEK293
	max_indelphi_freq_hct116	Max. prevalence predicted by inDelphi[14] for target deletion in HCT116
	max_indelphi_freq_k562	Max. prevalence predicted by inDelphi[14] for target deletion in K562
Visualization	cartoon	Cartoon showing the variant region with annotated µHs and cut sites
**Corresponding Guide file**		
IDs	id	Guide RNA ID number assigned by MHcut
	variant_id	Variant ID number assigned by MHcut
Guide RNA features	protospacer	Sequence of protospacer for Cas9 guide RNA
	mm0	Number of exact matches in the genome for the guide RNA
	m1_dist_1	Distance between cut site and perfect microhomology (left side) (bp)
	m1_dist_2	Distance between cut site and perfect microhomology (right side) (bp)
	mhdist_1	Distance between cut site and full microhomology (left side) (bp)
	mhdist_2	Distance between cut site and full microhomology (right side) (bp)
Predicted Prevalence of Target Deletion	indelphi_freq_mean	Prevalence predicted by inDelphi[14] for target deletion (mean of 5 cell types)
	indelphi_freq_mesc	Prevalence predicted by inDelphi[14] for target deletion in mESCs
	indelphi_freq_u2os	Prevalence predicted by inDelphi[14] for target deletion in U2OS cells
	indelphi_freq_hek293	Prevalence predicted by inDelphi[14] for target deletion in HEK293 cells
	indelphi_freq_hct116	Prevalence predicted by inDelphi[14] for target deletion in HCT116 cells

	indelphi_freq_k562	Prevalence predicted by inDelphi[14] for target deletion in K562 cells
Nested µHs	nb_nmh	Number of nested µHs for this guide RNA
	largest_nmh	µH length for the longest nested µH (bp)
	nmh_size	µH length of the "best" nested µH (bp)
	nmh_var_l	Deletion length of the "best" nested µH (bp)
	nmh_seq	Sequence of the "best" nested µH
	nmh_gc	GC content of the "best" nested µH
	nmh_score	Microhomology-Predictor[15] score for the highest scoring "best" nested µH

Supplementary Table 3. Plasmids used in this study

Purpose	Plasmid ID #	Plasmid name
CRISPR/Cas9	KW1193	pX-459v2_GLA-14bpDel
	KW1201	pX-459v2_ALAS2-4bpDel
	KW1299	pX-459v2_GLA-4bpDel-plus
	KW1300	pX-459v2_GLA-4bpDel-minus
	KW1301	pX-459v2_MECP2-9bpDel
	KW1302	pX-459v2_ADGRG1-15bpDel
	KW1303	pX-459v2_KDM6A-4bpDel
	KW1304	pX-459v2_FECH-5bpDel
	KW1306	pX-459v2_DYSF-5bpDel
Myogenic differentiation	KW698	PB-TAC-ERN-hMyoD
	KW158	pCAG-PBase

Supplementary Table 4. Primers used for sgRNA construction in this study

Target	Primer ID#	Primer Name	Sequence
GLA-14bpDel	dna2594	GLA-14bpDel-sgRNA-s	caccgTACCATGGGCTGGCTGCACT
	dna2595	GLA-14bpDel-sgRNA-as	aaacAGTGCAGCCAGCCCATGGTAc
ALAS2-4bpDel	dna2620	ALAS2-4bpDel-sgRNA-s	caccgCTTTGAGCTCATGAGTGAGT
	dna2621	ALAS2-4bpDel-sgRNA-as	aaacACTCACTCATGAGCTCAAAGc
GLA-4bpDel-plus	dna2868	GLA-4bpDel-plus-sgRNA-s	caccGGACGTAATTGCCATCAATC
	dna2869	GLA-4bpDel-plus-sgRNA-as	aaacGATTGATGGCAATTACGTCC
GLA-4bpDel-minus	dna2870	GLA-4bpDel-minus-sgRNA-s	caccGCCCAAGGGGTCCTGATTGA
	dna2871	GLA-4bpDel-minus-sgRNA-as	aaacTCAATCAGGACCCCTTGGGC
MECP2-9bpDel	dna2872	MECP2-9bpDel-sgRNA-s	caccGGCTTTTCCCTGGGGACTGT
	dna2873	MECP2-9bpDel-sgRNA-as	aaacACAGTCCCCAGGGAAAAGCC
ADGRG1-15bpDel	dna2874	ADGRG1-15bpDel-sgRNA-s	caccGCCCCTGGCAACCGTTGCCC
	dna2875	ADGRG1-15bpDel-sgRNA-as	aaacGGGCAACGGTTGCCAGGGGC
KDM6A-4bpDel	dna2876	KDM6A-4bpDel-sgRNA-s	caccgAAGGCTGGTAACACTGTTTG
	dna2877	KDM6A-4bpDel-sgRNA-as	aaacCAAACAGTGTTACCAGCCTTc
FECH-5bpDel	dna2878	FECH-5bpDel-sgRNA-s	caccGTGGTGGAGCAGCTGTACTG
	dna2879	FECH-5bpDel-sgRNA-as	aaacCAGTACAGCTGCTCCACCAC
DYSF-5bpDel	dna2882	DYSF-5bpDel-sgRNA-s	caccgAGGTGTTTGAGAACCAGACC
	dna2883	DYSF-5bpDel-sgRNA-as	aaacGGTCTGGTTCTCAAACACCTc

Lower-case characters indicate overhangs for BbsI cloning and 5'-G.

Supplementary Table 5. Synthetic crRNAs used for RNP formation in this study

Target	Sequence
GLA-14bpDel	TACCATGGGCTGGCTGCACT
ALAS2-4bpDel	CTTTGAGCTCATGAGTGAGT

GLA-4bpDel-plus	GGACGTAATTGCCATCAATC
GLA-4bpDel-minus	GCCCAAGGGGTCCTGATTGA
MECP2-9bpDel	GGCTTTTCCCTGGGGACTGT
ADGRG1-15bpDel	GCCCCTGGCAACCGTTGCCC
KDM6A-4bpDel	AAGGCTGGTAACACTGTTTG
FECH-5bpDel	GTGGTGGAGCAGCTGTACTG
DYSF-5bpDel	AGGTGTTTGAGAACCAGACC
COL1A-7bpDel	TTCTGTACGCAGGTGATTGG
KMT2D-8bpDel	GAGCCCGTCCAGGGGCTGTC
TTN-8bpDel	TGTACTTTGTGGTGGTCACT
PCNT-10bpDel	GGCGCCGCCCCGGCTGCCAG
NCF2-9bpDel	GGTGCTGGCAGCGGACAAGA
FLNA-13bpDel	CCCCATCAACATCTCAGAGA
SOX10-12bpDel	GTGGCGACAGGGCCCCCTTT
TSC2-18bpDel	GCTGGCGGAGCCGCTTGATG
RYR1-13bpDel	ACGAAGGATGCTGACATCTT
TSC2-16bpDel	AGGTAGGCTGGGGTTGGAGT
CCR5-32bpDel-Ye	CATACAGTCAGTATCAATTC
CCR5-32bpDel-He	CAGAATTGATACTGACTGTA

Supplementary Table 6. Primers used for sequencing of genomic DNA in this study

Target	Primer ID#	Primer Name	Sequence
GLA-14bpDel	dna2618	GLA-14bpDel-sequ-fw	CTTCTGGTATGGAAATAGGGCG
	dna2619	GLA-14bpDel-sequ-rev	AAGAAGGGTCTGAATAGAACCG
ALAS2-4bpDel	dna2666	ALAS2-4bpDel-sequ-fw	GGTTAGGGCTGACTCATTTCC
	dna2667	ALAS2-4bpDel-sequ-rev	TGAGGTATCTTGAAGGGTTGAG
GLA-4bpDel-plus/minus	dna2918	GLA-4bpDel-sequ-fw	TGGACATCTTTTAACCAGGAGAG
	dna2919	GLA-4bpDel-sequ-rev	AAAGTTGTCTCCCTGAAAAACC
MECP2-9bpDel	dna2920	MECP2-9bpDel-sequ-fw	ACTCCTTCACGGCTTTCTTTTTG
	dna2921	MECP2-9bpDel-sequ-rev	AGGGTGAGCAAAGGATTATCTTG
ADGRG1-15bpDel	dna2922	ADGRG1-15bpDel-sequ-fw	TCTCTCGAGAAATTGGAAGCTC
	dna2923	ADGRG1-15bpDel-sequ-rev	ACTGGCTACACCATCAATGC
KDM6A-4bpDel	dna2924	KDM6A-4bpDel-sequ-fw	AGTGTCGAGGGACTTCCTAATC
	dna2925	KDM6A-4bpDel-sequ-rev	TGTTTAAATCCCCAGAGCGAAG
FECH-5bpDel	dna2926	FECH-5bpDel-sequ-fw	AAGTTCACATACCAGCTGAAGAG
	dna2927	FECH-5bpDel-sequ-rev	TTAGTTTTACCAGCAACCAACTC
DYSF-5bpDel	dna2930	DYSF-5bpDel-sequ-fw	TTGGCATTCAACTCTGTGGTG
	dna2931	DYSF-5bpDel-sequ-rev	AAGATGCAACTTGACAACATACG
COL1A-7bpDel	dna3319	COL1A-7bpDel-sequ-fw	GTTTTTATTTCACTTGGCTTCAGCG
	dna3320	COL1A-7bpDel-sequ-rev	GATTGGGGGAGAAGAAACAAGAG
KMT2D-8bpDel	dna3323	KMT2D-8bpDel-sequ-fw	CTTTCTTCAAGGTAATACAGGTGG
	dna3322	KMT2D-8bpDel-sequ-rev	CTCCAGAGCTTCATGATTTGTTTG
TTN-8bpDel	dna3325	TTN-8bpDel-sequ-fw	GGGGATTATACCACCTATGCTTC
	dna3326	TTN-8bpDel-sequ-rev	GAACTGAGTAATCTTTGGTGGGG
PCNT-10bpDel	dna3323	PCNT-10bpDel-sequ-fw	TGCACGAAGTCAGCGACAG
	dna3324	PCNT-10bpDel-sequ-rev	CTTCACTGCGTACTGACACG
NCF2-9bpDel	dna3327	NCF2-9bpDel-sequ-fw	GGGAACTGATCCTTCAGGCAG
	dna3328	NCF2-9bpDel-sequ-rev	GCCCAGAAGGAGTTCTGTGTAG
FLNA-13bpDel	dna3329	FLNA-13bpDel-sequ-fw	AGATACTCCTGAATGGGGCTC
	dna3330	FLNA-13bpDel-sequ-rev	CAATGATAAACTCTGCAGGCTC
SOX10-12bpDel	dna3331	SOX10-12bpDel-sequ-fw	CATGTCAGACCCTCACTATCTGT
	dna3332	SOX10-12bpDel-sequ-rev	AGGTGAAGACAGAGACCGC
TSC2-18/16bpDel	dna3333	TSC2-18/16bpDel-sequ-fw	CAACTTTGTCCACGTGATCGTC
	dna3334	TSC2-18/16bpDel-sequ-rev	CAATCTGTGCCTCTATGTCTGTG

RYR1-13bpDel	dna3335	RYR1-13bpDel-sequ-fw	GCTGGAAACTCTAGACAGCCT
	dna3336	RYR1-13bpDel-sequ-rev	TCTTATAACTGGAGGTCTTGGAGA
CCR5-32bpDel-Ye/He	dna3292	CCR5-32bpDel-Ye/He-sequ-fw	CATGACTGACATCTACCTGCTC
	dna3284	CCR5-32bpDel-Ye/He-sequ-rev	GTCCAACCTGTTAGAGCTACTG

Supplementary Table 7. Primers used for qRT-PCR in this study

Target	Primer ID#	Primer Name	Sequence
ALAS2	dna3046	ALAS2_qPCR-fw	TGTCCGTCTGGTGTAGTAATGA
	dna3047	ALAS2_qPCR-rev	GCTCAAGCTCCACATGAAACT
FECH	dna3048	FECH_qPCR-fw	GGAGATGTTCACGACTTCCTTC
	dna3049	FECH_qPCR-rev	GAATGGTGCCAGCTTATTCTGA
GAPDH	dna878	Human GAPDH-F371-392	ACAACTTTGGTATCGTGGAAGG
	dna879	Human GAPDH-R471-453	GCCATCACGCCACAGTTTC
