# Supplementary material for: Genome-wide microhomologies enable precise template-free editing of biologically relevant deletion mutations
Source: Nat Commun. 2019 Oct 24;10:4856. doi: 10.1038/s41467-019-12829-8 (PMC6813315; doi:10.1038/s41467-019-12829-8)
Supplement: Supplementary file 3 — Reporting Summary [file 41467_2019_12829_MOESM3_ESM.pdf]

nature research

Corresponding author(s): NCOMMS-19-03896

Last updated by author(s): Sep 9, 2019

Reporting Summary

Nature Research wishes to improve the reproducibility of the work that we publish. This form provides structure for consistency and transparency in reporting. For further information on Nature Research policies, see Authors & Referees and the Editorial Policy Checklist .

Statistics

For all statistical analyses, confirm that the following items are present in the figure legend, table legend, main text, or Methods section.

n/a	Confirmed	
☐	☒	The exact sample size (*n*) for each experimental group/condition, given as a discrete number and unit of measurement
☐	☒	A statement on whether measurements were taken from distinct samples or whether the same sample was measured repeatedly
☐	☒	The statistical test(s) used AND whether they are one- or two-sided<br>*Only common tests should be described solely by name; describe more complex techniques in the Methods section.*
☒	☐	A description of all covariates tested
☒	☐	A description of any assumptions or corrections, such as tests of normality and adjustment for multiple comparisons
☐	☒	A full description of the statistical parameters including central tendency (e.g. means) or other basic estimates (e.g. regression coefficient) AND variation (e.g. standard deviation) or associated estimates of uncertainty (e.g. confidence intervals)
☒	☐	For null hypothesis testing, the test statistic (e.g. *F*, *t*, *r*) with confidence intervals, effect sizes, degrees of freedom and *P* value noted<br>*Give P values as exact values whenever suitable.*
☒	☐	For Bayesian analysis, information on the choice of priors and Markov chain Monte Carlo settings
☒	☐	For hierarchical and complex designs, identification of the appropriate level for tests and full reporting of outcomes
☒	☐	Estimates of effect sizes (e.g. Cohen's *d*, Pearson's *r*), indicating how they were calculated

Our web collection on statistics for biologists contains articles on many of the points above.

Software and code

Policy information about availability of computer code

Data collection	MHcut v1
Data analysis	Jellyfish v2.2.6<br>Microhomology-Predictor (Bae et al., 2014)<br>Gencode v28<br>R v1.0.153<br>Microsoft Excel for Mac v16.16.1<br>TIDE v2.01<br>PennCNV v1.0.3<br>Illumina GenomeStudio (2011.1 for v1.2 and 2.0.4 for v1.3)<br>GWASTools v1.2.R<br>MAD v1.0.1<br>GenomeJack (Mitsubishi Space Software)<br>BD FACS Diva Software v8.0.1<br>FlowJo v9.7.6 or higher

For manuscripts utilizing custom algorithms or software that are central to the research but not yet described in published literature, software must be made available to editors/reviewers. We strongly encourage code deposition in a community repository (e.g. GitHub). See the Nature Research guidelines for submitting code & software for further information.

Data

Policy information about availability of data

All manuscripts must include a data availability statement. This statement should provide the following information, where applicable:

- Accession codes, unique identifiers, or web links for publicly available datasets
- A list of figures that have associated raw data
- A description of any restrictions on data availability

MHcut is written in Python and available at the Python Package Index repository (PyPI) and https://github.com/WoltjenLab/MHcut. A Docker container is also provided. The results on the dbSNP and ClinVar deletions can be explored in the web application https://mhcut-browser.genap.ca/. The scripts from this analysis are available on the GitHub repository and the data was deposited at https://doi.org/10.6084/m9.figshare.9118364. The data that support the findings of this study are available from the corresponding author upon request.

Field-specific reporting

Please select the one below that is the best fit for your research. If you are not sure, read the appropriate sections before making your selection.

[x] Life sciences [ ] Behavioural & social sciences [ ] Ecological, evolutionary & environmental sciences

For a reference copy of the document with all sections, see nature.com/documents/nr-reporting-summary-flat.pdf

Life sciences study design

All studies must disclose on these points even when the disclosure is negative.

Sample size	Not relevant to this study
Data exclusions	No data was excluded
Replication	Three successful biological replicates
Randomization	Not relevant to this study
Blinding	Not relevant to this study

Reporting for specific materials, systems and methods

We require information from authors about some types of materials, experimental systems and methods used in many studies. Here, indicate whether each material, system or method listed is relevant to your study. If you are not sure if a list item applies to your research, read the appropriate section before selecting a response.

Materials & experimental systems

n/a	Involved in the study	
[ ]	[x]	Antibodies
[ ]	[x]	Eukaryotic cell lines
[x]	[ ]	Palaeontology
[x]	[ ]	Animals and other organisms
[x]	[ ]	Human research participants
[x]	[ ]	Clinical data

Methods

n/a	Involved in the study	
[x]	[ ]	ChIP-seq
[ ]	[x]	Flow cytometry
[x]	[ ]	MRI-based neuroimaging

Antibodies

Antibodies used	anti-human CD235a-FITC and CD71-APC (biolegend)<br>FITC and APC Mouse IgG2a antibodies (biolegend)<br>Nanog (D73G4) XP Rabbit mAb (Cell Signaling)<br>Purified Mouse Anti-Oct3/4 (BD Biosciences)<br>Alexa Fluor 546 goat anti-rabbit immunoglobulin G (IgG) (H+L) (life technologies)<br>Alexa Fluor 546 goat anti-mouse IgG (H+L) (invitrogen)
Validation	Validation conducted by providing company.

Eukaryotic cell lines

Policy information about cell lines

Cell line source(s)	1383D6 human iPSCs (CiRA, Japan)<br>H1 human ESCs (WA01) (WiCell, USA)<br>HEK293T cells (Thermo Scientific, USA)
Authentication	We performed SNP array for authentication of ES and iPS cell lines.<br>All the cell lines used were also authenticated by the providing companies and publications. Please refer to the following links.<br>H1 human ESCs (WiCell, USA): https://www.wicell.org/home/stem-cells/catalog-of-stem-cell-lines/wa01.cmsx<br>1383D6 human iPSCs (CiRA, Japan): https://www.sciencedirect.com/science/article/pii/S104620231530181X?via%3Dihub<br>HEK293T cells (Thermo Scientific, USA): https://dharmacon.horizondiscovery.com/uploadedFiles/Resources/tla-hek293t-cell-line-manual.pdf
Mycoplasma contamination	All cell lines tested negative for Mycoplasma contamination.
Commonly misidentified lines<br>(See ICLAC register)	*Name any commonly misidentified cell lines used in the study and provide a rationale for their use.*

Flow Cytometry

Plots

Confirm that:

- [x] The axis labels state the marker and fluorochrome used (e.g. CD4-FITC).
- [x] The axis scales are clearly visible. Include numbers along axes only for bottom left plot of group (a 'group' is an analysis of identical markers).
- [ ] All plots are contour plots with outliers or pseudocolor plots.
- [x] A numerical value for number of cells or percentage (with statistics) is provided.

Methodology

Sample preparation	For measurement of mCherry fluorescence intensities, 3.0 x 10^5 hiPSCs were resuspended in FACS buffer (PBS containing 2% FBS), filtered through a cell strainer and analyzed.<br>For the confirmation of erythroid differentiation, 1 x 10^5 cells were stained with anti-human CD235a-FITC (1:20) and CD71-APC (1:20) (biolegend) in a total volume of 10 µl FACS buffer containing 0.5 µl Human TruStain FcXTM (biolegend).
Instrument	BD LSRFortessa Cell Analyzer<br>BD FACS ARIA II Cell Sorter
Software	Data collection: BD FACS Diva Software version 8.0.1<br>Data analysis: FlowJo software v9.7.6
Cell population abundance	No sorting experiment was conducted.
Gating strategy	Preliminary FSC and SSC gates were set for doublet exclusion.

- [ ] Tick this box to confirm that a figure exemplifying the gating strategy is provided in the Supplementary Information.
